# Exploiting cell-mediated contraction and adhesion to structure tissues *in vitro*

**DOI:** 10.1098/rstb.2014.0200

**Published:** 2015-02-05

**Authors:** Uchena N. G. Wudebwe, Alistair Bannerman, Pola Goldberg-Oppenheimer, Jennifer Z. Paxton, Richard L. Williams, Liam M. Grover

**Affiliations:** 1 School of Chemical Engineering, University of Birmingham, Birmingham B15 2TT, UK; 2 School of Biomedical Sciences, University of Edinburgh, Edinburgh EH8 9AG, UK

**Keywords:** extracellular matrix, ligament, tendon, tissue engineering, collagen, ceramic

## Abstract

Progress in tissue engineering is now impacting beyond the field of regenerative medicine. Engineered tissues are now used as tools to evaluate the toxicity of compounds or even to enable the modelling of disease. While many of the materials that are used to facilitate tissue growth are designed to enable cell attachment, many researchers consider that the contraction and modification of these matrices by attached cells is not desirable and take measures to prevent this from occurring. Where substantial alignment of the molecules within tissues, however, is a feature of structure the process of contraction can be exploited to guide new matrix deposition. In this paper, we will demonstrate how we have used the cell contraction process to generate tissues with high levels of organization. The tissues that have been grown in the laboratory have been characterized using a suite of analytical techniques to demonstrate significant levels of matrix organization and mechanical behaviour analogous to natural tissues. This paper provides an overview of research that has been undertaken to determine how tissues have been grown *in vitro* with structuring from the molecular, right through to the macroscopic level.

## 1. Introduction

Adhesion is important at all levels of biology and is critical to both structure and function. At the molecular level, it is the adhesion of enzymes to their substrates that facilitates many of the chemical reactions that are vital to life. Beyond this scale, the attachment of cells to an extracellular matrix (ECM) controls how cells behave and can respond to their environment [1,2]. Subsequent levels of organization, facilitated through adhesion between molecules within the ECM, are what enable connective tissues to perform under demanding mechanical loads placed on them by the body [3]. This paper summarizes the adhesions that are important at each length scale and describes how adhesion is being exploited to regenerate tissues and recapitulate the impressive levels of organization. The main focus of the review is on highly aligned tissues, ligament and tendon, but the methods reported could be used to generate more complex structure. The review is organized so that the adhesive forces important at the ECM level, cell-matrix level and tissue level are discussed in turn.

## 2. The extracellular matrix: molecular nature and structural adaptation

The ECM is a three-dimensional space within an organ or tissue that surrounds populations of cells and functions as an adhesive and supportive structure. The ECM is formed from water, proteins, proteoglycans and glycosaminoglycans (GAGs), each of which have important mechanical and biological roles. It is the proportions and orientations of each of these components that determine tissue function and physical performance and any damage to this structure can have important physical and biological consequences, potentially resulting in long-term disability. In addition to playing an important structural role, the organization of these ECM components strongly influences cell behaviour, offering cues that control cell differentiation, attachment, recruitment, migration and further ECM production [4]. The interplay between the embedded cells and the ECM itself means that tissues are able to constantly remodel in response to changes in loading and oxygen levels so that they are able to respond to the demands that are placed on the body. This impressive ability to adapt also makes the regeneration of tissues to their native states challenging. Soft connective tissues contain high proportions of structural fibrillar proteins such as collagen types I and II with modifications in structure that enable them to exhibit impressive mechanical properties. Cartilage, for example, obtains its shock-absorbing properties from high concentrations of hyaluronan and proteoglycan aggrecans and the rigidity of bone is a function of calcium phosphate present in the fibrillar collagen matrix. Below, the principle roles of collagen, GAGs and proteoglycans are introduced in more detail. It is noted that other molecules within the ECM (elastin, laminin and vitronection) have important mechanical and biological functions, but these lay beyond the scope of this paper.

### (a) Collagen

**Figure 1.**
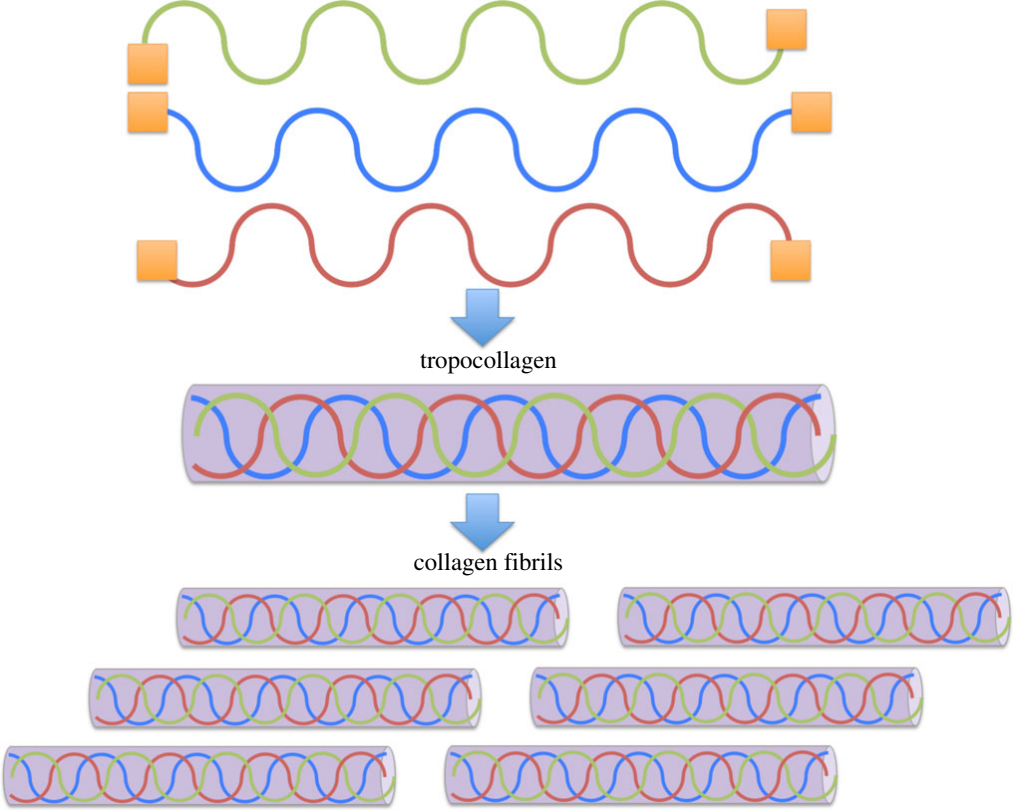
The assembly of collagen fibrils from tropocollagen molecules. Procollagen molecules are expressed within the cell and are modified via the cleavage of N and C termini. The resulting molecules self assemble to form tropocollagen molecules, which are subsequently self-assembled into collagen fibrils. Fibrillar diameter depends strongly on tissue type. (Online version in colour.)

Collagen is the most abundant protein that is found in the human body [5]. There are, at present, 28 known collagen types, which can be classified into fibril forming (I, II and XI), fibril associated (IX, XII and XIV), transmembrane (XIII and XVII), network forming (VI, VII and X), anchoring basement membrane (IV) and a selection of others with highly specific functions. In ligaments and tendons, type I is the most prevalent, constituting up to 90% of the total weight. Collagen type I is a structural protein with a triple helical conformation made up of two α1 chains and one α2 chain, that offers load-bearing tissue sufficient mechanical strength to withstand uniaxial and multiaxial loads [5]. The folding of the triple helix is due to glycine (Gly—the smallest amino acid) recurring in the Gly-X-Y amino acid sequence from which collagen is formed. X and Y are most commonly the amino acids proline and hydroxyproline, giving a repeating motif of Gly-Pro-Hyp [6]. It is the close packing of the three strands, hydrogen bonding and the high concentration of amino acids proline and hydroxyproline that stabilizes the triple helix [6]. The amino acids proline and 4-hydroxyproline are also referred to as imino acids [6,7] as they have one hydrogen atom attached to the nitrogen atom and when this forms part of an additional bond, there are no free hydrogen atoms available for hydrogen bonding. As a result, the presence of proline/hydroxyproline in the collagen molecule provides the flexibility needed to maintain the helical conformation for stability. When X and Y residues are not imino acids, they allow collagen fibril formation through attachment to other collagen molecules, integrins or ECM molecules [7,8]. Further stabilization of the collagen triple helix is achieved through binding of individual helices (tropocollagens) into collagen fibrils [9]. These fibrils can also join to form bundles and cross-link for enhanced stability [9] (figure 1).

### (b) Glycosaminoglycans

Glycosaminoglycans (GAGs) are linear polysaccharide chains, which are usually highly sulfated and negatively charged [10]. Sulfated GAGs include chondroitin, dermatan and keratan, whereas heparan, a basement membrane GAG and hyaluronan are unsulfated [11]. GAGs have extensive interaction with proteins such as growth factors, chemokines and cytokines [12] and the GAG long chains shield the proteins from proteolysis [13]. Connective tissues have high quantities of hyaluronan (hyaluronic acid), which aids cell migration and proliferation and maintains tissue hydration [14]. While the long chains are not very flexible, they are highly soluble in water and assume conformations that can trap large volumes of aqueous medium to form hydrated gels, a property that gives connective tissues their resistance to compressive stress [1].

### (c) Proteoglycans

Proteoglycans are GAGs that are covalently bonded to a protein and, as with GAGs, proteoglycans are important for signal transduction and also provide additional structural support to tissues [15]. Aggrecan, versican, neurocan and brevican, collectively known as lecticans, are members of the chondroitin sulfate proteoglycan family that offer resilience to compressive force [16]. Other small proteoglycans whose proteins have leucine-rich repeat structures (SLRPs), form u-shaped structures suitable for protein–protein interactions [17]. Members of the SLRP family are decorin, biglycan, fibromodulin, lumican and keratoca. Decorin is present in connective tissue and, along with other SLRPs, contributes to collagen fibril assembly and its regulation [17]. A study by Matuszewski *et al.* [18] with the human supraspinatus tendon, located in the shoulder, showed that aggregan and biglycan distribution varied throughout the tendon with the highest concentration being in the anterior and posterior regions that experience the highest compressive stress within this tissue and are prone to injury; decorin concentration was high throughout the tendon as expected in areas of high tensile strength. Proteoglycans that contain heparan sulfate can bind with growth factors and other signalling molecules that can accelerate cell proliferation and modulate behaviour [1].

**Figure 2.**
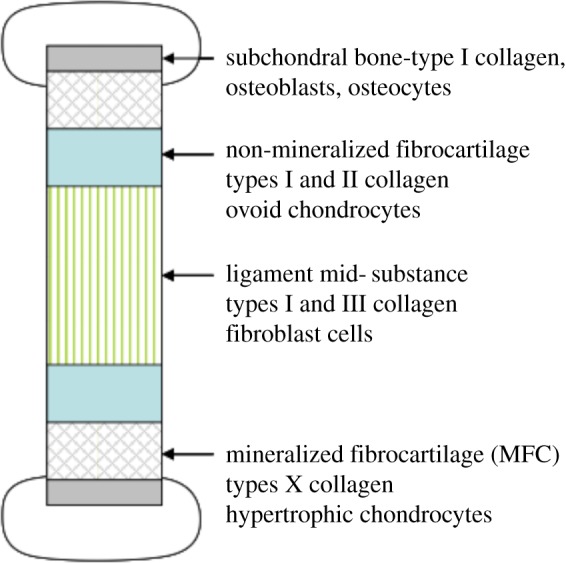
The heterogeneous structure of ligament tissue which prevents failure at the interface between bone and sinew. (Online version in colour.)

The distribution of matrix components through tissues is critical to mechanical performance. In the case of ligaments, the ligament midsubstance consists of collagen type I fibrils, which are aligned parallel to the principle axis of loading (figure 2). Moving towards the extremities of a ligament, there is a disruption in this order such that the collagenous matrix becomes richer in proteoglycan and GAG molecules with the formation of a homogeneous fibrocartilage structure. Directly adjacent to the bone, there is a region of mineralized fibrocartilage that directly abuts the subchondral bone. This exquisite level of structural adaptation means that the sinew is loaded almost purely in tension at its central portion and the mineralized region is placed under compression. This places each of the tissue components in a loading environment in which they may perform optimally and minimizes the chances of failure during locomotion [3]. It is this level of ordering which also provides a significant challenge within the field of regenerative medicine. It is, at present, impossible to reproduce these properties in a synthetic material; there is therefore a move towards using cells to create such structures outside the body for subsequent implantation. In order to understand how this may be done, it is important to understand how cells may interact with and be influenced by matrix components and other cells.

## 3. Cell–matrix interactions

**Figure 3.**
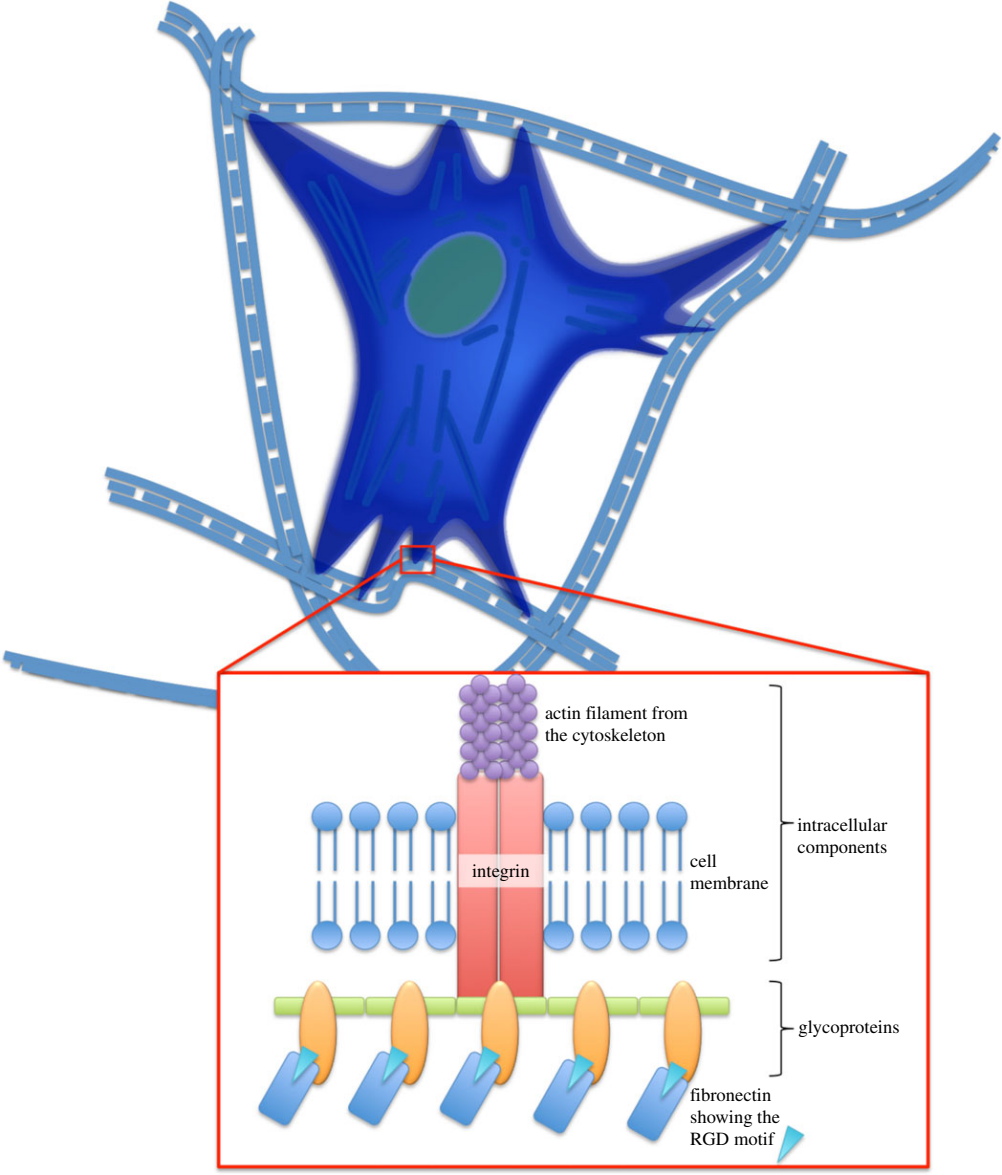
The interactions that occur between a cell adhesive fibrillar matrix bearing the RGD motif and an adhesion-dependent mammalian cell. The integrin subunits mediate attachment of the matrix to the actin cytoskeleton and enable stress transfer that can initiate tissue remodelling. (Online version in colour.)

Cells that are found within the ECM are critical to long-term adaptation and can therefore have an important role in the process of regeneration, particularly, in tissue deposition and restructuring. As the majority of human cells are anchorage dependent, their ability to form an adhesion with the ECM is critical to cell survival and behaviour [19]. The cell membrane consists of a phospholipid bilayer engulfed by a polysaccharide layer called the glycocalyx, which is ionizable resulting in a net negative charge on the cell surface [1]. The polymeric materials used for culturing cells *in vitro* are typically coated with poly-l-lysine, which has positively charged amine groups [1,20]. As a consequence, non-specific adhesion of various types of cells to solid surfaces occurs through weakly attractive electrostatic van der Waals forces [1]. The attachment of cells to the ECM is more specific and occurs through the interaction of moieties on ECM molecules with cell-surface glycoproteins known as integrins (figure 3). Integrin molecules contain 1–18 α and 1–8 β subunits arranged in different combinations. Many integrins bind to the same ligands on ECM components that contain the amino acid sequence Arg-Gly-Asp commonly known as RGD [1,21]. Typical ECM components that have the RGD sequence are vitronectin, bronectin and tenascin, though on collagen and laminin these are more obscure [22].

Fibroblasts express integrins with subunit arrangement α_1_β_1_ and α_2_β_1_ that bind to collagen simultaneously, however α_2_β_1_ has been found to have a greater affinity for collagen type I [23]. In three-dimensional collagen matrices, cells deficient in α_1_β_1_ receptors showed decreased growth, whereas presence of α_2_β_1_ enhanced the expression of collagen-degrading enzymes MMP-1 and MMP-13 [23,24], which mediates matrix renewal. The two integrins appear to have different roles because during wound healing α_2_β_1_ expression is upregulated by fibroblasts while that of α_1_β_1_ is downregulated [23]. Endothelial cells proliferate during wound healing and they possess α_V_β_3_ as their most prominent integrin [24]. In addition, dermal endothelial cells cultured in fibronectin or fibrin gels expressed higher mRNA levels of α_V_β_3_ than when cultured in collagen gels. As a result, α_V_β_3_ is an integrin implicated in cell–fibrin adhesion. Cell–cell adhesion can also be mediated by fibrinogen though binding of α_IIb_β_3_ and α_X_β_1_ on fibrinogen motifs Lys-Gln-Ala-Gly-Asp-Val (KQAGDV) and Gly-Pro-Arg-Pro (GPRP), respectively [25].

The clustering of integrins between a cell membrane and ECM molecules results in the formation of a focal adhesion complex, which anchors a network of actin stress fibres within the cell. This link between the cell and the ECM is what enables the cells to experience the mechanical loading applied to the cell population and critically activates pathways that enable the digestion of matrix and the concomitant deposition of new ECM. An improved understanding of the processes that occur during cell adhesion and how such processes govern tissue formation has opened the possibility of creating tissues *in vitro.* This may be achieved by placing the cell population in an environment that can stimulate the attached cells to secrete new ECM. Given the reported differences in cell behaviour when cultured in a three-dimensional structure as compared with in a monolayer system, much work has recently been dedicated to understanding the fundamental differences in behaviour between cell populations that are cultured in two and three dimensions. There is a now a general consensus that reproducing the complex tissue architectures that are found *in vivo* requires the provision of a three-dimensional support that provides cues to stimulate the embedded cell population to develop new matrix. The remainder of the paper will detail how three-dimensional culture in biologically derived matrices may enable the formation of organized tissue structures.

## 4. Tissue production *in vitro* exploiting cell–matrix interactions

**Figure 4.**
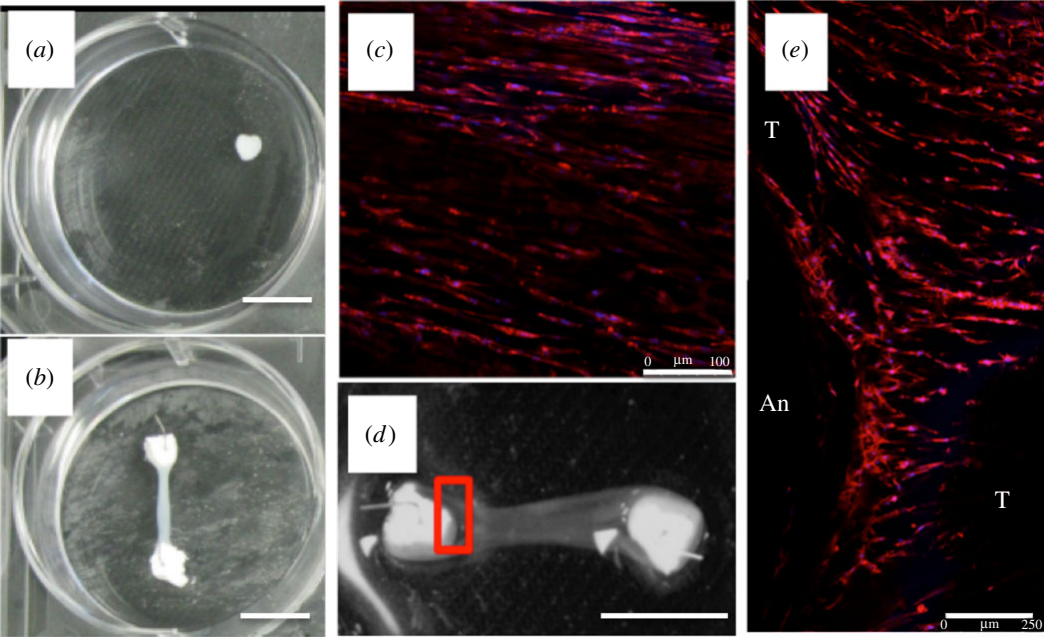
Free floating (*a*) and attached (*b*) fibrin-gel cultures seeded with fibroblasts after three weeks in culture (scale bars, 10 mm). (*c*) By 15 days of culture, cells in the central portion (stained with DAPI and phalloidin) were elongated and were orientated along the long axis of the sinew structure (scale bar, 100 μm). (*d*,*e*) Close examination of the interface with the ceramic brackets showed that levels of orientation varied from the ligament midsubstance (labelled T) to the interface with the anchor (labelled An). Scale bar, 8 mm in (*d*), 250 mm in (*e*). (Online version in colour.)

One way to encourage cells to secrete their own matrix and digest that to which they are attached is through the use of a temporary matrix to support the cells during growth. A matrix that has been shown to enable this is fibrin which facilitates cell attachment through an integrin matrix and may be readily reorganized by seeded cells. It is readily available through the collection of a small blood sample and fulfils an important haemostatic role during the process of wound healing in the body, providing a matrix onto which cells can attach and eventually be replaced by native tissue. It is formed from the cleavage of fibrinogen by thrombin, which results in the formation of fibrinopeptides A and B. It is the affinity of what are known as knobs A and B, which fit into holes a and b and result in gelation through the formation of protofibrils, which eventually self assemble to form a fibrillar network [26–28]. Fibroblasts seeded into a fibrin gel on the surface of a culture dish then associate with the fibrillar structure and will begin to contract the matrix and eventually a sphere of gelatinous material will be formed within the culture dish (figure 4*a*). By retaining the fibrin gel in the dish by using pins and sutures [29,30] or a calcium-phosphate ceramic with an inherently tissue-adhesive nature (figure 4*b*) [31,32], a gel cylinder is formed between the points of retention. The retention of the sinew-like structure by the ceramic brackets results in a passive tension between the ceramics [33] and this forces cell and molecular alignment [34] within the newly formed matrix (figure 4*c–f*). The cells have elongated cell bodies and nuclei suggestive of a tenocytic phenotype [35]. Interestingly, the level of orientation is significantly lower at the interface with the calcium phosphate ceramic suggestive of the development of a structure with multiple levels of organization (figure 4*e*).

**Figure 5.**
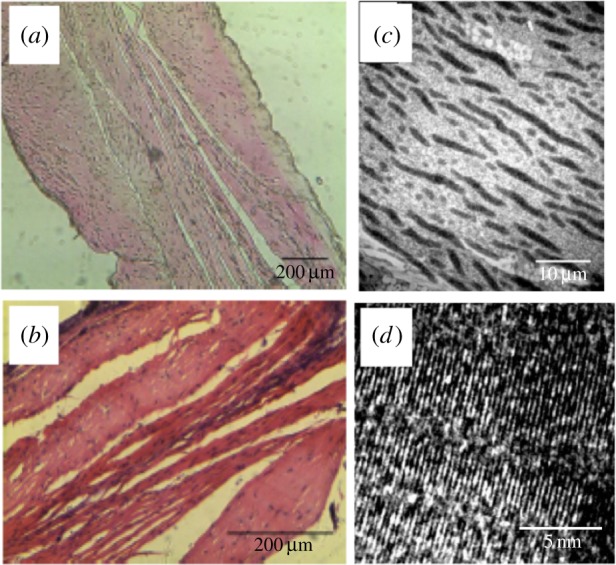
Imaging of ECM in the engineered tissues. Haematoxylin and eosin staining demonstrated the extent of cell and matrix alignment within the structure (*a*,*b*) and the enhanced density of the sinews following treatment with anabolic agents (*b*). Transmission electron microscopy demonstrated the presence of aligned fibrillar structures along the long axis of the sinew on the micro- (*c*) and nano-scales (*d*). (Online version in colour.)

After short periods of culture, the sinews typically consist of very small quantities of collagen (less than 5 wt%) [29,30]. However, it has been shown that it is possible to accelerate type I collagen deposition within the sinew by chemical treatment with ascorbic acid and proline [31], the former of which is involved in processing the procollagen molecules and the latter is added to prevent substrate limitation of collagen I production. Others have also demonstrated that it is possible to enhance collagen production through the application of a cocktail of growth factors [36]. It has also been demonstrated that intermittent bouts of cyclic mechanical conditioning of the sinew model results in an enhancement of collagen deposition and the mechanical strength of the resulting structures, although care must be taken to prevent matrix damage that can occur as a consequence of the application of more than 5% strain [37]. Recent unpublished results from our group have shown that it is possible to generate sinews with as high as 50 wt% of collagen I by also optimizing the mechanical properties of the supporting fibrin gel. Our hypothesis for this phenomenon is that an increase in local modulus enhances the mechanical stimulus provided to the cell population and upregulates collagen through the ERK pathway. Histological analysis demonstrated that the sinew itself had a highly orientated ECM (figure 5*a*) with the use of the collagen promotors ascorbic acid (AA) and proline (P) causing an enhancement in the density of the matrix (figure 5*b*). Examination of the ultrastructure using transmission electron microscopy (TEM) demonstrated the alignment of fibrils at the micro (figure 5*c*) and nano scales (figure 5*d*).

## 5. Structuring tissue on the macroscopic scale

**Figure 6.**
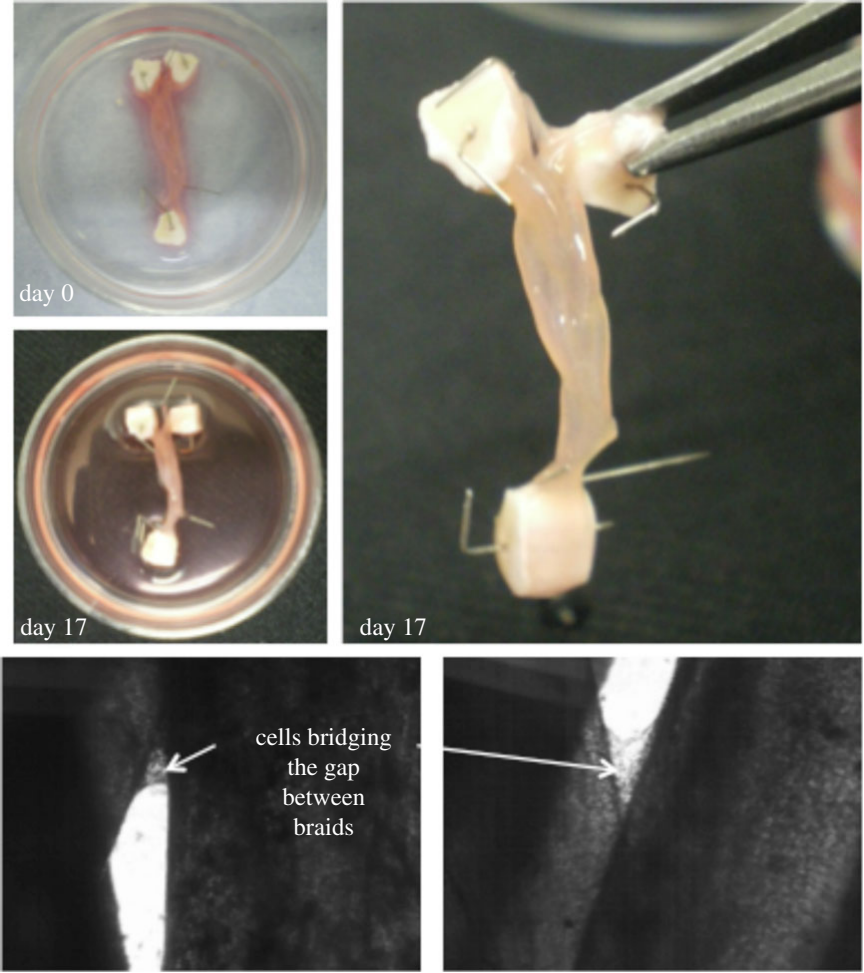
Macrostructral organization of the engineered sinews demonstrating the amalgamation of the individual strands over a period of 17 days in culture. (Online version in colour.)

Ligaments and tendons are formed from multiple fascicles that interact with one another. The interactions that form between individual fascicles have been proposed to be responsible for enhancing the mechanical properties exhibited by native ligaments and tendon. The fascicles themselves are bundled together and surrounded by a membranous structure known as the epitenon [38]. In order to replicate this arrangement, it is possible to culture individual sinews in close proximity over a period of weeks. In the course of this time, the individual strands grow together and eventually amalgamate. The amalgamation is as a consequence of cellular bridging between the structures in addition to the formation of ECM between the individual strands (figure 6). Importantly, this kind of macrostructural organization of the sinew-like structures results in mechanical augmentation of the structure and deformation behaviour similar to that observed for native sinews.

## 6. Conclusion and future directions

By controlling how cells are able to manipulate a soft matrix *in vitro* it is possible to generate a heterogeneously structured material with structure and deformation characteristics that resemble those of native ligaments and tendons. The mechanical properties that are exhibited by these structures are orders of magnitude lower than those exhibited by native ligaments and tendons and so it is unlikely that they will replace current methods for ligament and tendon reconstruction in the very near future. Current directions of research are seeking to use this structured material as a means to augment healing in the body. We have recently published a method for decelularizing these structures [39] so that they may be manufactured and stored prior to implantation into patients to enhance the success of currently existing methods of tissue reconstruction. The aligned collagenous matrix is also being used as a model for diseases where mineral deposition within the soft tissues is a debilitating side effect.
